# Systematic Review and Meta-analysis of Suprascapular Notch Morphological Variability: Do We Know Everything?

**DOI:** 10.7759/cureus.55852

**Published:** 2024-03-09

**Authors:** George Tsakotos, Răzvan C Tudose, George Triantafyllou, Christos Koutserimpas, Mugurel C Rusu, Dimitrios Flevas, Maria Piagkou

**Affiliations:** 1 Department of Anatomy, School of Medicine, Faculty of Health Sciences, National and Kapodistrian University of Athens, Athens, GRC; 2 Department of Anatomy, Faculty of Dentistry, “Carol Davila” University of Medicine and Pharmacy, Bucharest, ROU; 3 Department of Orthopedics and Traumatology, "251" Hellenic Air Force General Hospital of Athens, Athens, GRC; 4 Department of Orthopedics, Metropolitan Hospital, Athens, GRC

**Keywords:** suprascapular nerve entrapment, anatomical variations, morphology, morphological variability, suprascapular notch

## Abstract

The suprascapular notch represents a depression on the lateral part of the superior border of the scapula, medially to the coracoid process. The current paper presents a systematic review with a meta-analysis of the suprascapular notch morphological variability. Related clinical implications were further discussed as well to emphasize the value of the topic. A total of 31 articles were included in the meta-analysis, which depicted great heterogeneity. Thus, due to the different classification systems, difficulties were faced in creating a complete and united classification. All the problems and pitfalls that arise from each classification system were discussed, and we concluded with the most complete one. The knowledge of the suprascapular notch morphological anatomy is of great importance, especially for orthopedic surgeons, due to its relationship with the suprascapular nerve. Thus, further research in this area is adequate.

## Introduction and background

The suprascapular notch is a depression on the lateral part of the superior border of the scapula, medially to the coracoid process. The notch is transformed into a foramen after the superior transverse scapular ligament complete ossification. According to classical anatomy textbooks, the suprascapular artery runs above the ligament, and the homonymous vein and nerve course below the ligament. The suprascapular notch is the main compression site for the suprascapular nerve [1]. The suprascapular notch variant morphology may be an important risk factor for the nerve’s compression; thus, knowledge of its morphology is essential for the nerve’s surgical decompression [2]. Rengachary et al. [3], Natsis et al. [1], and Polguj et al. [4] studies have investigated the suprascapular notch morphology using different classification systems. Tubbs et al. [5] described different shapes of the suprascapular notch, such as the U-shaped, the V-shaped, and the notch absence, as well as the superior transverse scapular ligament’s partial or complete ossification. Polguj et al. [4] emphasized the suprascapular notch morphometric details' value by suggesting the following three factors for consideration: the notch maximum depth, and the superior and middle transverse diameters. All classification systems are summarized in Table 1.

**Table 1:** The classification systems of the suprascapular notch morphology. MD, maximum depth; MTD, middle transverse diameter; STD, superior transverse diameter

Classification	Type	Characterization
Natsis et al. (2007) [1]	I	Without a discrete notch
	II	Notch with greater transverse than vertical diameter (U-shaped)
	III	Notch with greater vertical than transverse diameter (V-shaped)
	IV	Suprascapular foramen
	V	Suprascapular notch and foramen
Rengachary et al. (1979) [3]	I	Wide depression of the scapula superior border
	II	V-shaped notch
	III	U-shaped notch
	IV	Small V-shaped notch
	V	U-shaped with partial ossification of the ligament medial part
	VI	Completely ossified ligament with foramen
Polguj et al. (2011) [4]	IA	MD>STD and STD
	IB	MD>STD and STD=MTD
	IC	MD>STD and STD>MTD
	II	MD=STD=MTD
	IIIA	MD
	IIIB	MD
	IIIC	MDMTD
	IV	Suprascapular foramen
	V	Without a discrete notch

The current systematic review with meta-analysis summarizes the suprascapular notch morphology according to the available classification systems. The possible clinical implications are further discussed.

## Review

Materials and methods

Search Strategy

**Figure 1:**
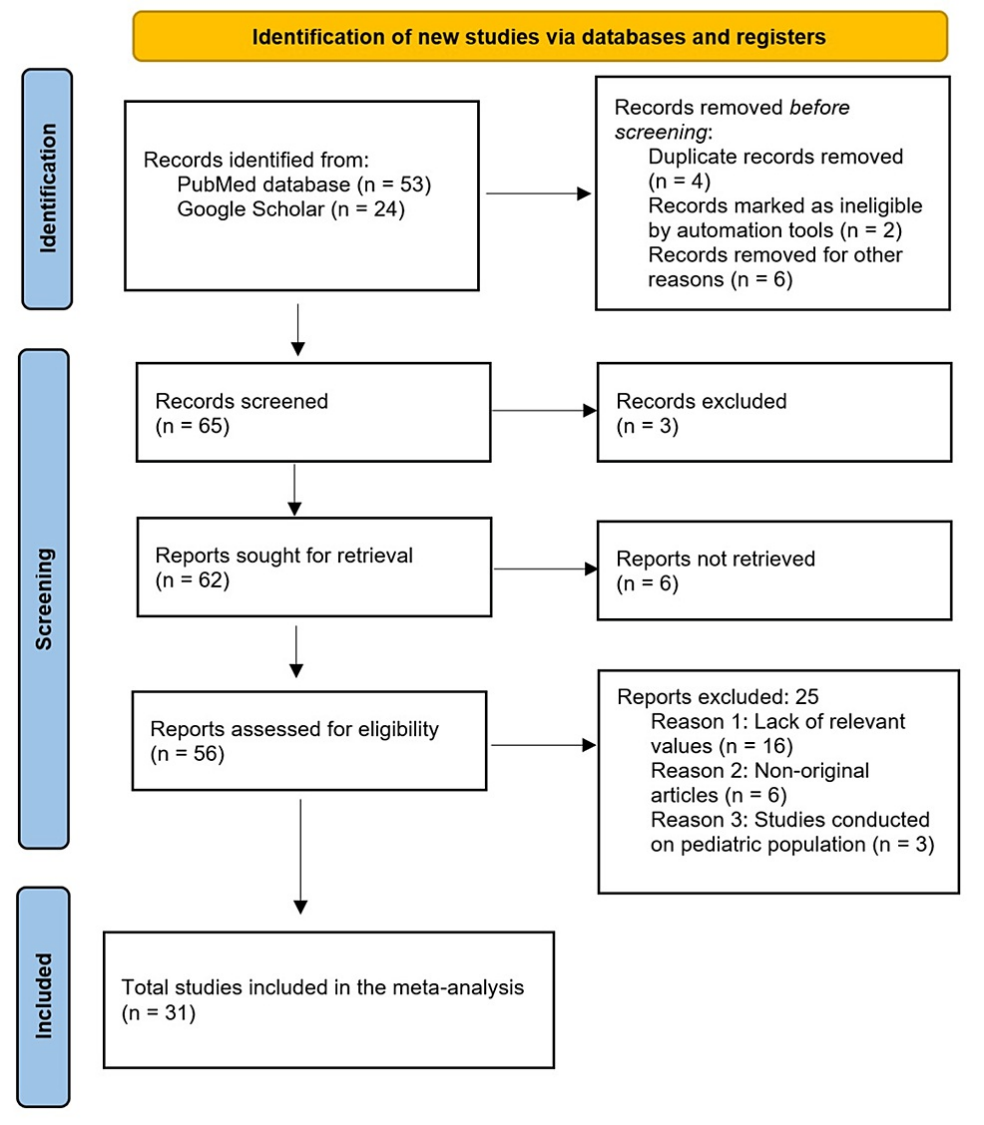
PRISMA flow diagram. PRISMA, Preferred Reporting Items for Systematic Reviews and Meta-Analyses

Following the Preferred Reporting Items for Systematic Reviews and Meta-Analysis (PRISMA) guidelines [6] (Figure 1), a meticulous systematic search was conducted across the electronic databases PubMed and Google Scholar. The objective was to identify all articles pertinent to the suprascapular notch variants, up to September 2023. The search strategy combined terms of the notch anatomy with its morphological variants, such as "suprascapular notch" AND "anatomical variant" AND "morphology" AND "morphological variability” AND "anatomy," with different combinations. Additionally, manual searching was implemented within the reference lists of selected articles and relevant reviews to ensure a comprehensive inclusion of studies.

Eligibility Criteria

Inclusion criteria comprised the following: (1) explicitly reported on the suprascapular notch variants, (2) delineated data on the count of specific variants, and (3) specified the total number of evaluated cases or samples. Exclusion criteria comprised the following: (1) non-original research articles (reviews, case reports, letters, or commentaries), (2) studies lacking transparent and extractable data on specific variants, (3) studies focusing exclusively on pediatric populations, and (4) studies with a relatively small sample (n<50).

Data Extraction

Two independent investigators executed the data extraction, focusing on the authorship, publication year, total case count, registration of each suprascapular notch variant, and the methodological or classification employed. In instances of discrepancies, consensus was reached through discussion, and, if required, a third investigator was consulted for arbitration. Studies that reported zero or no data were excluded to ensure the model’s validity, as such entries could not provide meaningful variance and would compromise the reliability of the regression estimates. In the study by Raj et al. [7], due to a discrepancy between the number of type 2 suprascapular notches in the main text and the table, we relied on the data summarized in the table, since that value aligned with the corresponding percentages. In the study by Soni et al. [8], the authors employed two distinct classification systems: Natsis's classification and a shape-based classification. In the process of data collection and selection for the present meta-analysis, the studies of Polguj et al. [9] and Podgorski et al. [10] were ultimately excluded due to methodological limitations. Polguj et al. [9] did not provide sufficient data to be reliably included in the meta-analysis, and Podgorski et al. [10] failed to completely classify their data according to the Polguj et al. [9] classification system, making it incompatible with the other included studies. These exclusions were made to maintain the analysis’ integrity and robustness, ensuring that only studies meeting the methodological criteria were incorporated.

Quality Assessment and Risk of Bias

Using the Newcastle-Ottawa Scale (NOS) for case-control studies, each article underwent rigorous quality appraisal. To address the unique requirements of our prevalence analysis, item 3 from the "Selection" category was excluded. Studies were assigned a quality score ranging between 0 and 7. Only those studies achieving a threshold score of 4 or higher were incorporated into the meta-analysis (Table 2).

**Table 2:** Studies included in the analysis. NOS for case-control studies. NOS, Newcastle-Ottawa Scale

Author	Method	Population	Total number of cases	NOS scale
Natsis et al. (2007) [1]	Natsis	Greek	423	4
Rengachary et al. (1979) [3]	Rengachary	American	226	7
Polguj et al. (2011) [4]	Polguj	Polish	86	6
Raj et al. (2019) [7]	Natsis	Indian	250	5
Soni et al. (2012) [8]	Natsis	Indian	100	4
Adewale et al. (2020) [11]	Rengachary	Ugandan	50	5
Agrawal et al. (2014) [12]	Unclassified	Indian	293	6
Agrawal et al. (2015) [13]	Unclassified	Indian	728	6
Ahmed (2018) [14]	Polguj	Egyptian	65	4
Albino et al. (2013) [15]	Rengachary	Italian	500	4
Benia et al. (2023) [16]	Unclassified	Uruguayan	62	7
Daripelli et al. (2020) [17]	Rengachary	Indian	200	5
Emad et al. (2017) [18]	Natsis	Egyptian	100	6
Inoue et al. (2014) [19]	Rengachary	Japanese	762	5
Inoue et al. (2021) [20]	Rengachary	Japanese	552	5
Iqbal et al. (2009) [21]	Unclassified	Indian	250	4
Jamwal et al. (2018) [22]	Rengachary	Indian	91	5
Kannan et al. (2014) [23]	Rengachary	Indian	400	6
Khalkho et al. (2018) [24]	Rengachary	Indian	69	6
Kumar et al. (2014) [25]	Natsis	Indian	248	5
Mahdy and Shehab (2013) [26]	Natsis	Egyptian	132	5
Nayak and Gujar (2020) [27]	Rengachary	Indian	525	5
Polguj et al. (2013) [28]	Polguj	Polish	616	7
Sangam et al. (2013) [29]	Rengachary	Indian	104	5
Toneva and Nikolova (2014) [30]	Unclassified	Bulgarian	102	5
Ukoha et al. (2022) [31]	Rengachary	Nigerian	193	5
Vyas et al. (2012) [32]	Polguj	Indian	300	5
Wang et al. (2011) [33]	Natsis	Chinese	295	5
Yamakado (2016) [34]	Rengachary	Japanese	760	6
Yang et al. (2012) [35]	Unclassified	Korean	103	7
Zhang et al. (2019) [36]	Unclassified	Chinese	308	4

*Prevalence Analysis* 

The numerical values for each variant were gathered in an Excel table. For each incorporated study, the variant's prevalence was ascertained using the formula: Prevalence = number of specific variant cases / total studied cases. The standard error (SE) associated with each prevalence was computed as SE = sqrt(p(1-p)/n), where p was the prevalence of the specific variant and n was the total case count.

Statistical Analysis

In the current meta-analysis, Jamovi (version 2.3.21.0) and R software (version 4.1.2) were used, incorporating the “metaphor” library in R for specific calculations. Key configurations for the present meta-analysis were as follows:

Effect size selection: The effect size was used to understand how widespread variants were in the included studies. These effect sizes were reported as percentages, along with SEs to indicate their accuracy. We set Cohen’s d equivalence bounds at -0.5 and 0.5 to capture medium differences in anatomical research, which are significant in our field.

Model estimator: The authors used the DerSimonian-Laird random-effects model for our analysis due to the expected variability among the included studies.

Moderator type: The current analysis involved a mixed-effects model with a categorical moderator. This moderator helped us account for different methods used in studies for classifying the variants. In Jamovi, we faced some limitations, and, hence, some parts of this analysis were specifically carried out in R. The first category of the moderator was used as a reference, and others were compared against it.

Confidence interval (CI): We set a 95% CI to ensure the precision and reliability of our effect size and moderator estimates. This approach ensured our analysis was thorough, accurate, and unbiased.

Handling of Heterogeneity

Given the inherent heterogeneity among the included studies, primarily due to different methodologies, we initiated a subgroup analysis. We employed the I² statistic as a quantitative measure to assess heterogeneity levels within each subgroup.

Classification Systems

To ensure consistency in analysis, we categorized each study based on the classification system used. Each classification system was assigned a unique numeric identifier as follows: unclassified methodologies were referred to as the “unclassified method,” Natsis et al. [1] classification as the “Natsis method,” and Rengachary et al. [3] classification as the “Rengachary method.”

Polguj et al. Classification

The Polguj et al. [4] classification, known for its detailed morphological categorization, was treated distinctly, and referred to as the “Polguj method.” Four articles employing this classification were included. Given that these studies exclusively used the Polguj et al. [4] classification, the introduction of a categorical moderator was deemed unnecessary. This approach was adopted to maintain analytical integrity and clarity, eliminating the need to account for potential heterogeneity stemming from different classification methods.

Special Cases

Suprascapular foramen and notch: We chose not to include a moderator variable in our analysis of the suprascapular foramen and notch variant due to limited data. Only three studies have used the same classification system for this variant. Consequently, we calculated the pooled effect size to provide a consolidated measure of its prevalence.

J-shaped and double foramen variants: The “J-shaped” and "double foramina" variants were only represented in two studies. Due to statistical reliability and model convergence constraints, it was not possible to conduct a formal meta-analysis. Therefore, descriptive statistics were used to summarize the available data.

Type IC and IIIC variants in the “Polguj method”: Only three studies have identified the variants classified as type IC and IIIC within the Polguj et al. [4] classification system. Due to the limited datasets, it was difficult to carry out a reliable sensitivity analysis. Therefore, similar to the “suprascapular foramen and notch,” we concentrated on creating pooled effect sizes for these variants without using a moderator variable.

“Natsis method” exclusions: None of the studies included in the analysis of the variants “incomplete ossification” and “small V-shaped” utilized the classification system proposed by Natsis et al. [1]. Additionally, these analyses were carried out without the use of a categorical moderator to guarantee that the statistical reliability and convergence of the models were not compromised.

Assessment of Publication Bias

Potential publication bias was assessed for using funnel plots and supported by Egger’s regression test for plot asymmetry. Any detected asymmetry in the funnel plot or statistical significance of the regression test was indicative of potential publication bias.

Sensitivity Analysis

To ensure the credibility of our findings, we conducted a sensitivity analysis. This included omitting individual studies one by one, followed by a recalculation of the pooled estimate, to ensure that no single study had an excessive impact on the overall results.

Results

Search Synthesis

A total of 31 articles were ultimately included (Table 2). These selected articles provide a robust foundation for synthesizing existing knowledge on the subject and were instrumental in achieving the objectives of our study.

Prevalence of the Suprascapular Notch Variants

Table 3 and Table 4 summarize the prevalence rates for each variant across the included studies. This foundational information establishes the relative commonality or rarity of each variant in the studied population.

**Table 3:** Table with prevalence for articles employing the unclassified, Natsis, and Rengachary methods. ND, no data available; SS, suprascapular; SSN, suprascapular notch

Author	Absent SSN (%)	U-shaped (%)	V-shaped (%)	SS foramen (%)	SS foramen and notch (%)	Incomplete ossification (%)	Small V-shaped (%)	J-shaped (%)	Double foramen (%)
Natsis et al. (2007) [1]	8.27	41.84	41.84	7.32	0.71	ND	ND	ND	ND
Rengachary et al. (1979) [3]	7.96	48.23	30.97	3.98	ND	5.75	3.10	ND	ND
Raj et al. (2019) [7]	2.00	76.00	20.40	1.60	ND	ND	ND	ND	ND
Soni et al. (2012) [8]	5.00	72.00	20.00	3.00	ND	ND	ND	ND	ND
Adewale et al. (2020) [11]	14.00	50.00	12.00	8.00	ND	4.00	12.00	ND	ND
Agrawal et al. (2014) [12]	13.65	45.05	23.55	ND	ND	ND	17.75	ND	ND
Agrawal et al. (2015) [13]	4.25	26.37	52.60	ND	ND	ND	3.84	ND	ND
Albino et al. (2013) [15]	12.40	22.80	19.80	3.60	ND	10.20	31.00	ND	ND
Benia et al. (2023) [16]	8.06	ND	ND	6.45	ND	16.13	ND	ND	ND
Daripelli et al. (2020) [17]	27.50	30	22.5	10.00	ND	4.50	5.50	ND	ND
Emad et al. (2017) [18]	10.00	43.00	39.00	5.00	3.00	ND	ND	23.00	ND
Inoue et al. (2014) [19]	11.41	30.05	23.50	4.33	ND	15.88	14.83	ND	ND
Inoue et al. (2021) [20]	20.47	34.42	18.30	3.62	ND	9.42	13.76	ND	ND
Iqbal et al. (2009) [21]	18.00	13.20	20.00	ND	ND	ND	ND	22.00	ND
Jamwal et al. (2018) [22]	21.97	64.83	1.10	6.60	ND	4.40	1.10	ND	ND
Kannan et al. (2014) [23]	20.00	52.00	10.00	10.00	ND	4.00	4.00	ND	ND
Khalkho et al. (2018) [24]	10.14	36.23	34.78	4.34	ND	7.24	7.24	ND	ND
Kumar et al. (2014) [25]	35.08	54.03	8.46	10.48	ND	ND	ND	ND	ND
Mahdy and Shehab (2013) [26]	6.06	45.45	43.94	3.03	1.51	ND	ND	ND	ND
Nayak and Gujar (2020) [27]	28.00	47.04	12.00	8.95	ND	1.90	2.10	ND	ND
Sangam et al. (2013) [29]	21.15	59.61	8.65	1.92	ND	5.77	2.88	ND	ND
Toneva and Nikolova (2014) [30]	15.68	25.5	44.11	2.94	ND	11.76	ND	ND	ND
Ukoha et al. (2022) [31]	3.10	70.46	22.28	ND	ND	3.10	1.03	ND	ND
Wang et al. (2011) [33]	9.50	57.96	28.13	4.06	ND	ND	ND	ND	0.33
Yamakado (2016) [34]	8.29	39.47	24.86	3.55	ND	5.13	18.68	ND	ND
Yang et al. (2012) [35]	4.85	ND	ND	3.88	ND	ND	ND	ND	ND
Zhang et al. (2019) [36]	ND	48.37	6.16	2.92	ND	1.94	ND	ND	0.65

**Table 4:** Table with prevalence for articles employing the Polguj method. ND, no data available

Author	Type IA (%)	Type IB (%)	Type IC (%)	Type II (%)	Type IIIA (%)	Type IIIB (%)	Type IIIC (%)	Type IV (%)	Type V (%)
Polguj et al. (2011) [4]	15.10	3.50	5.80	2.30	8.20	2.30	44.20	7.00	11.60
Ahmed (2018) [14]	15.38	24.61	ND	1.54	21.53	26.15	ND	3.08	7.69
Polguj et al. (2013) [28]	14.12	3.08	6.98	1.95	2.92	0.97	52.27	4.72	12.99
Vyas et al. (2012) [32]	6.00	5.00	9.33	2.67	2.33	2.67	37.67	3.67	30.67

*Mixed-Effects Model Results* 

Table 5 shows a multi-faceted overview of the prevalence of various variants and the potential impact of the different methods on these prevalences. The ensuing analysis elucidates these effect sizes, the associated significance levels, and the ripple effect of different classification methods. The salient findings are as follows. In terms of the suprascapular notch absence (10.578%), approximately 10.6% of the population was identified with no notch. The high confidence, as indicated by the robust p<0.001, suggests that this prevalence is likely to be a true reflection within the broader population. This morphology did not have a significant difference between the different methods. In terms of the U-shaped suprascapular notch (31.7%), a pronounced 31.7% of this sample exhibited this variant. Its prevalent representation, backed by the significant p<0.001, emphasizes its common occurrence. This variant was statistically more frequent with the Natsis method (p=0.003). The V-shaped suprascapular notch (28.981%) represented a substantial 29% of the sample. This variant is undeniably significant within the sampled cohort, a fact echoed by the p<0.001. The suprascapular foramen (3.62%) was presented in 3.6% of the sample, with a significant p=0.007. This variant did not have statistical differences between the different methods. Incomplete ossification, Small V-shaped, and specific types from the “Polguj method,” no moderators were applied, stemming from methodological uniformity across the included studies for those precise variants. The clear demarcation and significance of these results accentuate the influence of the variant classifications and emphasize the pivotal role of methodological selection in research outcomes. The J-shaped suprascapular notch was examined based on data from two distinct studies by Emad et al. [18] and Iqbal et al. [21]. Despite employing different methodologies - the “Natsis method” in Emad et al.’s study [18] and the “unclassified method” in Iqbal et al.’s study [21] - the studies reported remarkably similar prevalence rates at 23% and 22%, respectively. This consistency across different methods lends preliminary support to the idea that the prevalence of the J-shaped variant may be relatively stable. However, it is important to approach these findings with caution. The number of studies available for this particular anatomical variant is limited, preventing us from conducting a more robust meta-analysis. For the double suprascapular foramen, two studies were sourced for analysis. Wang et al. [33], utilizing the “Natsis method,” found a single case among 295 samples, yielding a prevalence of approximately 0.34%. Zhang et al. [36], employing the “unclassified method,” identified two cases out of 308 samples, translating to a 0.65% prevalence. Though the two studies used different classification methods, the prevalence rates reported are both very low, falling below 1%. This suggests that the double foramen variant is relatively rare. However, given the limited number of studies and their methodological differences, these results should be interpreted with caution.

**Table 5:** Mixed-effects model results. A p-value less than 0.05 was considered significant. CI, confidence interval; N/A, not available; SE, standard error;; SS, suprascapular; SSN, suprascapular notch

Anatomical variant	Number of studies	Estimate (%)	SE	95% CI (lower bound – upper bound)	P-value	Moderator estimate (decimal)		SE (Moderator)		Moderator p-value	
						Moderator 1	Moderator 2	Moderator 1	Moderator 2	Moderator 1	Moderator 2
Absent SSN	26	10.578	0.0308	4.5 – 16.6	<0.001	-0.00109	0.0502	0.0417	0.0374	0.979	0.179
U-shaped SSN	25	31.7	0.0615	19.6 – 43.7	<0.001	0.242	0.130	0.0809	0.0726	0.003	0.072
V-shaped SSN	25	28.981	0.0612	17.0 – 41.0	<0.001	-0.00404	-0.1065	0.0804	0.0720	0.960	0.139
SS foramen	23	3.62	0.0135	1.0 – 6.3	0.007	0.0103	0.0168	0.0165	0.0153	0.531	0.273
SS foramen and notch	3	0.942	0.00397	0.2 – 1.7	0.018	N/A		N/A		N/A	
Incomplete ossification	16	6.49	0.0105	4.4 – 8.5	<0.001	N/A		N/A		N/A	
Small V-shaped	15	9.04	0.0173	5.6 – 12.4	<0.001	N/A		N/A		N/A	
J-shaped	2	N/A	N/A	N/A	N/A	N/A		N/A		N/A	
Double foramen	2	N/A	N/A	N/A	N/A	N/A		N/A		N/A	
“Polguj method” classification											
Type IA	4	12.1	0.0287	6.5 – 17.7	<0.001	N/A		N/A		N/A	
Type IB	4	5.79	0.019	2.1 – 9.5	0.002	N/A		N/A		N/A	
Type IC	3	7.42	0.00827	5.8 – 9.0	<0.001	N/A		N/A		N/A	
Type II	4	2.1	0.00439	1.2 – 3.0	<0.001	N/A		N/A		N/A	
Type IIIA	4	5.3	0.0171	1.9 – 8.7	0.002	N/A		N/A		N/A	
Type IIIB	4	3.72	0.0167	0.5 – 7.0	0.026	N/A		N/A		N/A	
Type IIIC	3	44.9	0.0538	34.3 – 55.4	<0.001	N/A		N/A		N/A	
Type IV	4	4.35	0.00624	3.1 – 5.6	<0.001	N/A		N/A		N/A	
Type V	4	15.8	0.0477	6.5 – 25.2	<0.001	N/A		N/A		N/A	

Assessment of Heterogeneity and Publication Bias 

Table 6 presents valuable insights into the heterogeneity and publication bias metrics of the examined suprascapular notch morphological variants. The “absent suprascapular notch variant” exhibits a significant study variation with a pronounced value of I² (94.02%). The U-shaped suprascapular notch variant displays high heterogeneity (I²=96.03%), while the V-shaped suprascapular notch variant exhibits the highest heterogeneity (I²=97.58%). This, along with Egger's regression of 2.209 (p=0.027), raises concerns about the potential for publication bias. The suprascapular foramen variant also shows significant heterogeneity (I²=72.23%) and an Egger's regression of 2.655 (p=0.008), indicating potential publication bias issues. Other variants, including the suprascapular foramen and notch and certain classifications from the Polguj method, show varying levels of heterogeneity without strong indications of publication bias, as reflected in their respective Egger's regression p-values. Overall, the increased heterogeneity observed in the absent suprascapular notch, U-shaped, and V-shaped variants underscores the decision to employ a random-effects model for the analysis. The noteworthy p-values from Egger's regression for the absent suprascapular notch, V-shaped suprascapular notch, and suprascapular foramen variants highlight the importance of being mindful of potential publication bias in the meta-analysis.

**Table 6:** Heterogeneity and publication bias metrics. A p-value less than 0.05 was considered significant. SS, suprascapular; SSN, suprascapular notch

Variant	I² (%)	Egger's regression	P-value (Egger's regression)
Absent SSN	94.02	2.944	0.003
U-shaped	96.03	1.460	0.144
V-shaped	97.58	2.209	0.027
SS foramen	72.23	2.655	0.008
SS foramen and notch	2.93	1.412	0.158
Incomplete ossification	90.09	1.895	0.058
Small V-shaped	96.91	2.337	0.019
J-shaped	N/A	N/A	N/A
Double foramen	N/A	N/A	N/A
“Polguj method” classification			
Type IA	85.03	0.877	0.381
Type IB	82.53	3.270	0.001
Type IC	0	-0.153	0.879
Type II	0	0.110	0.913
Type IIIA	82.24	3.807	<0.001
Type IIIB	87.49	3.985	<0.001
Type IIIC	89.04	-0.320	0.749
Type IV	0	0.211	0.833
Type V	92.97	-0.203	0.839

Sensitivity Analysis and Observations

Table 7 offers detailed insights into the influence of individual studies on the results of our meta-analysis, especially concerning durability. Table 7 reinforces the need for thorough sensitivity analyses in meta-studies to discern the weight and influence of individual research contributions.

**Table 7:** Noteworthy observations and sensitivity analysis. *Cook’s distance (range) (others) refers to the range in Cook’s distance for the other analyzed studies.

Anatomical variant	Noteworthy studies	Cook's distance	Cook’s distance (others) (range)*	Original estimate (%)	Sensitivity analysis: estimate (%)	Original I² (%)	Sensitivity analysis: I² (%)	Observations/Comments
Absent SSN	Kumar et al. (2014) [25]	2.0	0 – 0.3	10.7	10.75	100	100	No substantial impact on heterogeneity or central tendency despite high Cook’s distance.
U-shaped	Iqbal et al. (2009) [21]	0.6	0 – 0.4	31.7	36.4	96.03	95.68	Major changes in effect size, indicating study impact.
V-shaped	Agrawal et al. (2015) [12]	1.1	0 – 0.8	28.981	22.64	97.58	96.34	1.24% change in heterogeneity; significant effect size adjustment.
SS foramen	Kumar et al. (2014) [25]	0.7	0 – 0.6	3.62	3.58	72.23	68.18	Minor changes in both effect size and heterogeneity, suggesting low influence of this study.
SS foramen and notch	Natsis et al. (2007) [1]	6	0 – 1	0.942	N/A	2.93	N/A	Unable to perform sensitivity analysis due to high Cook's distance and a limited number of studies. Interpretation of this variant's effect size and heterogeneity should be done with caution.
Incomplete ossification	Inoue et al. (2014) [20]	0.8	0 – 0.2	6.49	5.56	90.09	81.87	Moderate changes in both effect size and I², indicating study influence.
Small V-shaped	Albino et al. (2013) [15]	0.9	0 – 0.2	9.04	7.43	96.91	95.58	Significant changes in effect size and slight I² reduction, indicating study influence.
J-shaped	N/A	N/A	N/A	N/A	N/A	N/A	N/A	Insufficient data to perform a sensitivity analysis.
Double foramen	N/A	N/A	N/A	N/A	N/A	N/A	N/A	Insufficient data to perform a sensitivity analysis.
“Polguj method” classification								
Type IA	Vyas et al. (2012) [32]	0.6	0 – 0.1	12.1	14.3	85.03	0	Significant change in heterogeneity to 0% and a minor increase in effect size, indicating a notable influence of this study on overall results.
Type IB	Polguj et al. (2013) [28]	2.2	0.5 – 1.5	5.79	8.65	82.53	85.66	A moderate increase in both effect size and heterogeneity upon sensitivity analysis, suggesting that this study has a noteworthy influence on the overall meta-analysis.
Type IC	N/A	N/A	N/A	N/A	N/A	N/A	N/A	Insufficient data to perform a sensitivity analysis.
Type II	Polguj et al. (2013) [28]	0.35	0 – 0.15	2.1	2.35	0	0	Minimal change in both effect size and heterogeneity upon sensitivity analysis, indicating that this study has a negligible influence on the overall meta-analysis.
Type IIIA	Vyas et al. (2012) [32]	6	0.2 – 1.2	5.3	9.57	82.24	87.31	Marked variations in effect size and heterogeneity were observed after sensitivity analysis, signifying that this study's strong impact on the overall results of the meta-analysis.
Type IIIB	Polguj et al. (2013) [28]	4.5	0 – 2.5	3.72	7.2	87.49	89.1	The significant change in effect size highlights the study's substantial influence.
Type IIIC	N/A	N/A	N/A	N/A	N/A	N/A	N/A	Insufficient data to perform a sensitivity analysis.
Type IV	Polguj et al. (2013) [28]	0.5	0 – 0.3	4.35	3.93	0	0	A relatively stable but minor impact on the overall meta-analysis.
Type V	Vyas et al. (2012) [32]	0.7	0 – 0.3	15.8	12.0	92.97	10.37	The heterogeneity dropped dramatically, implying that this study was a significant source of variability in the original meta-analysis.

Discussion

The objective of this meta-analysis was to provide a comprehensive assessment of the variability in suprascapular notch morphology. However, certain challenges were encountered during the analysis of the included studies. Upon careful examination, significant heterogeneity was observed, likely due to the application of different classification systems. The current literature could not be standardized due to the existence of three distinct classification systems. The prevalence rates derived from the classification systems of Rengachary et al. [3] and Natsis et al. [1], which are currently the most used, are presented in Table 1. The classification system of Polguj et al. [4] was not evaluated in conjunction with the other two, and thus its pooled prevalence is presented separately in Table 4. Given these findings, which classification system offers the most comprehensive and precise results?

The Rengachary et al. (1979) classification system

In 1979, Rengachary et al. [3] conducted an analysis of dried and cadaveric scapulae and proposed a straightforward classification system. Type I encompassed a broad depression of the entire superior scapular border or the absence of the suprascapular notch. Types II and III were characterized by a V-shaped and U-shaped suprascapular notch, respectively. Type IV was similar to type II, but with a smaller V-shaped notch. Types V and VI denoted incomplete or complete ossification of the superior transverse scapular ligament.

The Natsis et al. (2007) classification system

According to the findings of Natsis et al. [1], a revised classification was proposed after analyzing 423 dried scapulae. In this system, type I indicated the suprascapular notch absence, while types II and III were based on the measurement of the vertical and transverse diameters of the notch and represented V-shaped and U-shaped notches, respectively. Types IV and V were related to the suprascapular foramen and the presence of both the foramen and notch.

The Polguj et al. (2011) classification system

**Figure 2:**
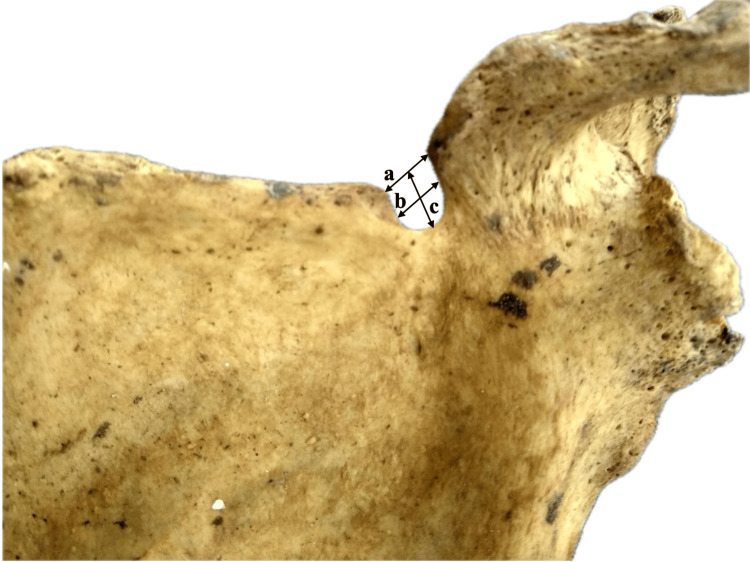
The suprascapular notch, according to the Polguj et al. classification. a, superior transverse diameter; b, middle transverse diameter; c, maximal depth [4]

**Figure 3:**
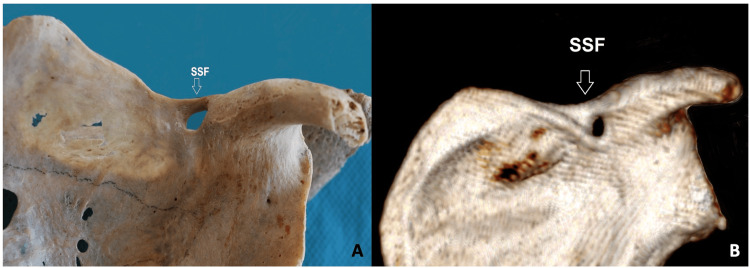
The SSF variant. A. On a dry scapula. B. On a computed tomography scapula. SSF, suprascapular foramen

Polguj et al. [4] suggested a different way of classifying the suprascapular notch based on its shape. They measured three diameters of the notch: its maximum depth, the superior transverse diameter, and the middle transverse diameter (Figure 2). They classified the suprascapular notch into five types. Type I is when the notch's maximum depth is larger than its superior transverse diameter. They further classified it based on the relationship between the superior and middle transverse diameters. Type II is when all three diameters are the same. Type III is the opposite of type I, where the superior transverse diameter is greater than the maximum depth. They again classified it based on the relationship between the superior and middle transverse diameters. Type IV is when there is a suprascapular foramen (Figure 3). Type V is when there is no suprascapular notch at all. Although the Polguj et al. [4] classification is the most complete, it is not commonly used because it is complicated.

The Debate Among the Classification Systems

Rengachary et al. [3] and Natsis et al. [1] classification systems are the most used in the current literature, probably due to their simplicity. Rengachary et al. [3] suprascapular notch of types II (V-shaped) and III (U-shaped) was a pitfall for researchers, which was partially corrected by the types II and III of Natsis et al. [1] classification, which proposed the measurements of the transverse and vertical diameter of the notch. However, Polguj types I, II, and III cover every possible shape. Secondly, the superior transverse scapular ligament incomplete ossification raises major confusion. Rengachary type V represented the “incomplete ossification of the superior transverse scapular ligament,” which was classified by the others. This type cannot be named as “incomplete ossification of the superior transverse scapular ligament” because it cannot be proved that this is an ossification of a suprascapular notch rather than a different shape, and that is why Polguj et al. [4] have classified those cases into types I, II, and III. It is important to mention that the three classification systems agreed on the suprascapular notch absence or absence of a discrete notch, and the suprascapular foramen. Hence, for the other types, the Polguj et al. [4] classification covers every possible shape and represents the most well-defined classification.

Clinical Significance

The morphology of the suprascapular notch is of great clinical interest to orthopedic surgeons as suprascapular nerve entrapment is a common neuropathy where the nerve is compressed, usually at the level of the suprascapular notch. The suprascapular nerve is a peripheral nerve responsible for both motor and sensory functions. Its motor signals are supplied to the supraspinatus and infraspinatus muscles, while its sensory branches cover the coracohumeral and coracoacromial ligaments, as well as the glenohumeral joint. Athletes who engage in overhead activities are frequently affected by primary suprascapular nerve entrapment syndrome. Excessive stretching of the suprascapular nerve at the suprascapular notch leads to posterior shoulder discomfort, accompanied by muscle weakness and wasting of the supraspinatus and infraspinatus muscles. Diagnosis of this condition is made by physical examination and imaging techniques, including magnetic resonance imaging, electromyography, and nerve conduction velocity tests. The suprascapular notch shape is a predisposing factor for suprascapular nerve entrapment. The small V-shaped notch is the most significant variant that can cause nerve entrapment. A narrow notch coexisting with an anomalous superior transverse scapular ligament may result in constriction, making it a risk factor for suprascapular neuropathy. Complete ossification of the suprascapular foramen occurs in a small percentage of cases, and it is still unclear if this variant should be included among the risk factors for suprascapular nerve entrapment. However, incomplete or complete ossification of the ligament may lead to traction-type injury of the nerve, according to Rengachary et al. [3,37]. Rare variants, such as double suprascapular foramen or the suprascapular nerve coexisting with suprascapular foramen, are also possible but less common. The development of suprascapular nerve entrapment is a multifaceted issue influenced by various factors, including the configuration of the notch, the ligament morphology, the existence of an enlarged suprascapular muscle, a fully ossified ligament, and the presence of a spinoglenoidal ligament. Suprascapular neuropathy can be challenging to diagnose due to its nonspecific symptoms, especially when muscle atrophy in the supraspinatus and infraspinatus muscles is present [38]. The effectiveness of surgical interventions is impacted by the length of time between symptom onset and surgery, as well as the root cause of nerve compression. Hence, investigating variations in structures within the suprascapular region can offer valuable insights to enhance the diagnosis and treatment of this medical condition [39].

Limitations

Our meta-analysis focused on the “Suprascapular foramen and notch” variant, but we only had access to three studies, which limited the robustness of the current analysis. We urge caution in interpreting the results due to this small sample size. We were unable to fully assess the impact of individual studies on the pooled effect size, which makes our findings less stable and interpretable. This highlights the need for further research on this variant to obtain more robust meta-analytic conclusions in the future. Our analysis has a limitation related to the “incomplete ossification” and “small V-shaped” variants. All the studies used in this analysis only used two classification methods, which makes it difficult to apply a categorical moderator in the analysis for this specific variant. This limits our ability to explore potential sources of heterogeneity and generalizes our findings to other studies that use different methods. To overcome this constraint, future research should use different classification systems, such as the Natsis classification, to obtain a more nuanced understanding of the factors influencing the prevalence of this anatomical variant. We were unable to conduct a meta-analysis for the J-shaped and double foramina variants of the suprascapular notch due to the limited number of studies available, which is a significant drawback. This limitation restricts our ability to provide a comprehensive view of the prevalence of these variants. Thus, further research is necessary to obtain a more robust analysis and a better understanding of their prevalence. However, this meta-analysis provides a detailed description of all the anatomical variants of the suprascapular notch, which can provide valuable insights for the diagnostic and therapeutic approach to the suprascapular nerve entrapment. We have performed a thorough description of all morphological variants so far reported in the literature and have highlighted the need for further research to associate the anatomical variations with the pathophysiology of the suprascapular nerve entrapment, as well as toward an easy-to-use and more accepted classification system of the suprascapular nerve morphology.

## Conclusions

This systematic review with meta-analysis aims to summarize the different suprascapular notch morphological variants. The study identifies both usual and unusual morphological variants of the notch. However, the current literature does not provide a well-defined classification system for such variability. Therefore, further research based on the findings of this review is essential. The knowledge of suprascapular notch morphology is particularly crucial for orthopedic surgeons as it is related to suprascapular nerve entrapment syndrome.
